# Global analysis of lysine 2-hydroxyisobutyrylation in wheat root

**DOI:** 10.1038/s41598-021-85879-y

**Published:** 2021-03-18

**Authors:** Feng Bo, Li Shengdong, Wang Zongshuai, Cao Fang, Wang Zheng, Gao Chunhua, Li Geng, Kong Ling’an

**Affiliations:** 1 Crop Research Institute, Shandong Academy of Agricultural Sciences, Ji’nan, Shandong 250100 People’s Republic of China; 2 College of Agronomy, Shandong Agricultural University, Tai’an, Shandong 271018 People’s Republic of China; 3 Cotton Research Center, Shandong Academy of Agricultural Sciences, Ji’nan, Shandong 250100 People’s Republic of China

**Keywords:** Plant development, Proteomics, Plant sciences

## Abstract

Lysine 2-hydroxyisobutyrylation (Khib) is a novel naturally occurring post-translational modification. The system Khib identification at proteomics level has been performed in various species and tissues to characterize the role of Khib in biological activities. However, the study of Khib in plant species is relatively less. In the present study, the first plant root tissues lysine 2-hydroxyisobutyrylome analysis was performed in wheat with antibody immunoprecipitation affinity, high resolution mass spectrometry-based proteomics and bioinformatics analysis. In total, 6328 Khib sites in 2186 proteins were repeatedly identified in three replicates. These Khib proteins showed a wide subcellular location distribution. Function and pathways characterization of these Khib proteins indicated that many cellular functions and metabolism pathways were potentially affected by this modification. Protein and amino acid metabolism related process may be regulated by Khib, especially ribosome activities and proteins biosynthesis process. Carbohydrate metabolism and energy production related processes including glycolysis/gluconeogenesis, TCA cycle and oxidative phosphorylation pathways were also affected by Khib modification. Besides, root sulfur assimilation and transformation related enzymes exhibited Khib modification. Our work illustrated the potential regulation role of Khib in wheat root physiology and biology, which could be used as a useful reference for Khib study in plant root.

## Introduction

Various protein post-translational modifications (PTMs) play important roles in diverse cellular processes and metabolism pathways regulation^1,2^. To data, more than 400 PTMs have been discovered in various living organisms as the result of fast developing high-resolution mass spectrometry (MS) based proteomics^3–6^. Lysine 2-hydroxyisobutyrylation (Khib) is a novel naturally occurring PTM which was firstly discovered in histone^2,7^. Its role in chromatin activities and gene transcription has been well illustrated^2,7^. Other cellular and biological processes could also be affected by Khib^8^.

Lysine 2-hydroxyisobutylome have been performed in some species to illustrate the mechanisms of Khib regulated diverse biological processes and cellular activitis. It has been reported that there are 1458 Khib sites in 369 proteins in *Saccharomyces cerevisiae* and 4735 Khib sites in 1051 proteins in *P. mirabilis*^9,10^. Moreover, a comparative 2-hydroxyisobutyrylome was performed in *E. coli*^11^. These studies have shown Khib was involved in various metabolism pathways and cellular components including glycometabolism, protein metabolism and ribosome in microorganism^9–11^. In animals, it was reported that Khib proteins were distributed in multiple cell components and participated in diverse biological processes control and adjustment in parasite *T. gondii*^12^. In addition, thousands of Khib sites and proteins were identified in various mammal cell lines including HeLa cells, mouse embryonic fibroblast (MEF) cells, Drosophila S2 cells, A549 cells, 5637 cells, HEK293T cells and Pluripotent Stem Cells^6,8,10,13–15^.

In plant, the study of Khib in plants is relative limited and the reported plant species is too less, only rice and *Physcomitrella* patens have been reported^13,14^. In rice, using developing seeds as material, 9916 Khib sites in 2512 proteins were identified^13^. Functional characterization analysis of these Khib proteins indicated various material and energy metabolism pathways were involved, such as glycolysis, gluconeogenesis, TCA cycle and some other material metabolism pathways^13^. In *Physcomitrella*, 11,976 Khib sites in 3001 proteins were screened, which involved in diverse molecular functions, cellular processes, and metabolism pathways^14^.

Bread wheat (*Triticum aestivum *L.) is one of the world’s most important food crops which serves as the staple food source for 30% world population^15^. Thus it is of great significance to study the mechanisms of wheat growth and development regulation, as well as wheat physiology. Previous studies have shown some PTMs, such as phosphorylation, acetylation and succinylation played a role in wheat physiology^16–18^. However, the roles of Khib in wheat growth and development and wheat physiology haven’t been reported. In addition, previous PTMs studies at proteomics level in wheat were mainly focused on the aerial parts, especially on leaves^16–18^. The studies of physiological roles of PTMs in wheat root tissues are relatively less.

In the present study, using fresh wheat root as material, the qualitative 2-hydroxyisobutyrylome was performed to screen Khib sites and proteins in wheat tissues as well as to illustrate the potential regulatory roles of Khib in wheat root growth and development. To data, our study is the first 2-hydroxyisobutyrylome study in high plant root tissues, which may shed light on the elucidation of the regulatory role of Khib in root physiology and biology.

## Results

### Detection of Khib in wheat root tissues

**Figure 1:**
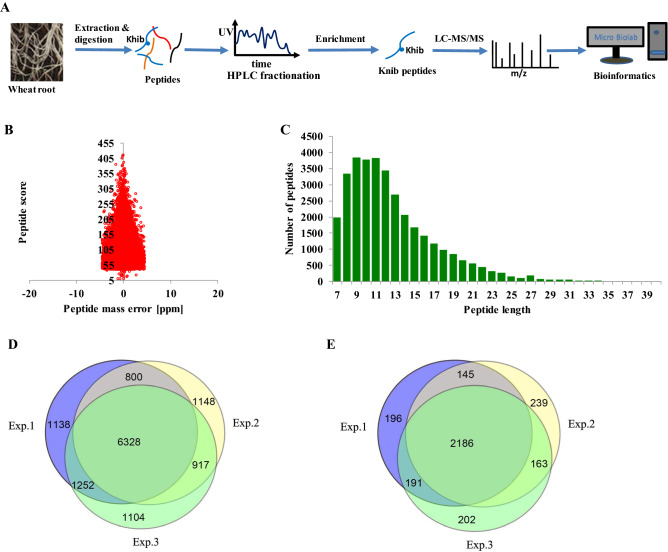
Systematic analysis of Khib in wheat roots. (**A**) Workflow for global Khib screening in wheat roots. (**B**) Mass error distribution of all the Khib peptides. (**C**) Peptide length distribution of all the Khib peptides. (**D**) Venn diagram of the identified Khib sites. (**E**) Venn diagram of the identified Khib proteins.

To comprehensively understand the role of Khib in plant root growth and development, the qualitative lysine 2-hydroxyisobutyrylome analysis was performed in wheat root tissues. As shown in Fig. 1A, after 40 days outdoor nature climate cultivation, wheat tissues were collected. Protein were extracted and digested with trypsin. Then the HPLC fractionation and affinity enrichment procedure were performed to obtain Khib peptides. After LC–MS/MS data acquisition and database searching, the Khib peptides and proteins were detected. Lastly, bioinformatics tools were used to characterize the modified peptides and proteins.

Quality control of MS data is the foundation for various proteomics based biological studies. As shown in Fig. 1B and C, the distributions of mass errors of the identified Khib peptides were less than 5 ppm, which had fitted to the requirements of MS accuracy. In accordance with the property of tryptic peptides, the majority peptides lengths ranged from 7 to 25 amino acids. The sample preparation and MS data have met the technically requirements. Totally, 12,687 Khib sites corresponding to 3322 proteins were detected, among which 6328 Khib sites corresponding to 2186 proteins were repeatedly identified (Fig. 1D,E) in three replicates, which indicated many proteins in wheat root growth and development were Khib modified. The detailed information of theses detected Khib peptides and proteins could be referred to supplementary information (Table S1).

To validate the wide distribution of Khib in wheat root tissues, a western blotting experiment were performed with pananti-2-hydroxyisobutyryllysine antibody (Fig. S1). Multiple protein bands of various sizes and smears were observed in western blotting; suggesting protein Khib is ubiquitous in wheat root tissues, which is consistent with the detected large number of Khib sites and proteins in MS data.

An in vitro assay was performed to explore where whether proteins in wheat root will be altered in 2-hydroxyisobutyrulation level by 3-Hydroxyisobutyryl-CoA. As shown in (Fig. S2), the 3-Hydroxyisobutyryl-CoA in vitro treated wheat root proteins haven’t shown significantly difference with the control at 2-hydroxyisobutyrulation level, implying 3-Hydroxyisobutyryl-CoA produces little influence on protein 2-hydroxyisobutyrulation in wheat root.

### Function classification and subcellular location prediction

**Figure 2:**
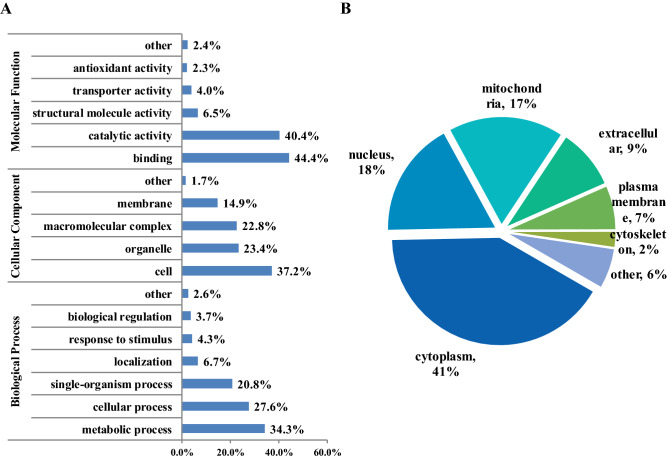
Functional classification and subcellular location analyses of the Khib proteins. (**A**) Function classification based on GO annotation. (**B**) Subcellular location from WoLF PSORT.

Gene ontology (GO)-based function analysis were conducted in the level of biological process, cellular component and molecular function to illustrate the role of Khib in wheat root morphogenesis and root physiology (Fig. 2A). In molecular function level, most Khib proteins were classified into binding and catalytic activity, whose percentage was 44.4% and 40.4%, respectively. Other molecular function related groups were also observed, such as structural molecule activity (6.5%), transporter activity (4.0%) and antioxidant activity (2.3%) while their ratios were very low. In the category of cellular component, the Khib proteins showed a wide cellular component distribution and the involved cellular components included cell (37.2%), organelle (23.4%), macromolecular complex (22.8%), and membrane (14.9%). As to the biological process, the majority of these Khib proteins were metabolic process, cellular process and single-organism process related, whose percentage were 34.2%, 27.6% and 20.8%, respectively. Moreover, localization (6.7%), stimulus response (4.3%) and growth regulation (3.7%) related proteins were detected as well.

Subcellular prediction analysis was carried out to decipher the intra-cell apartments where Khib proteins located within cell (Fig. 2B). The result showed that cytoplasm was the most preferred subcellular location where Khib protein appeared because as much as 41% Khib protein were localized to cytoplasm. The proportions of nucleus and mitochondrion located Khib proteins were almost the same with the percentage of 18% and 17%, respectively. In addition, extracellular component and plasma membrane located Khib proteins were relatively less, which accounted for only 9% and 7%, respectively. We also noticed that a few Khib proteins were distributed at cytoskeleton (2%). The rest Khib proteins were localized to other subcellular locations whose ratio was 6%.

The aforementioned analysis and results indicated that Khib proteins exhibited a wide biological function and subcellular location distribution in wheat root. In addition, binding and catalytic activity, metabolic and cellular processes were the primary biological events which protein Khib participated in and cytoplasm was the major subcellular apartment where protein Khib occurred.

### GO and KEGG pathway enrichment analysis

To further elucidate the nature of preferred target proteins of Khib, the enrichment analysis were implemented on the category of GO and KEGG pathway.

**Figure 3:**
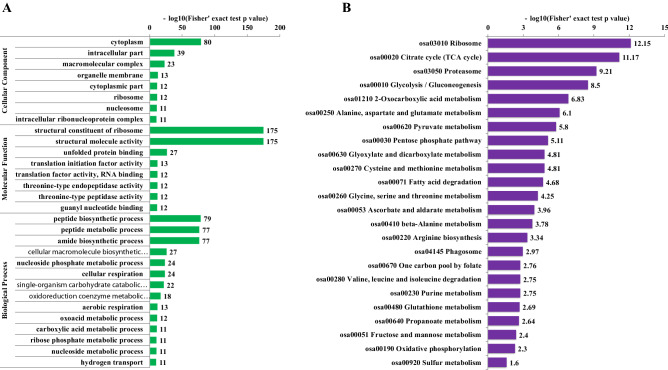
Enrichment analyses of the Khib proteins. (**A**) GO annotation based enrichment analysis. (**B**) KEGG pathway based enrichment analysis.

In the GO based enrichment analysis (Fig. 3A), cytoplasm was the top significantly enriched cellular component, then is intracellular part and macromolecular complex. Other cellular components were also significantly enriched including organelle membrane, ribosome and nucleosome. The cellular component enrichment analysis result was consist with subcellular location prediction result (Fig. 2B), which suggested Khib proteins were distributed in various subcellular locations or components among which cytoplasm was the first preferred subcellular place. In the molecular function level, the majorities dramatically enriched terms were ribosome, translation factors and peptidase activity related, implying protein metabolism including both protein synthesis and protein degradation is possibly influenced by Khib modification. Correspondingly, the top two markedly enriched peptide biosynthetic processes and peptide metabolic process in biological process analysis further consolidated this assumption. A number of carbon metabolism, respiration and energy production related biological processes were dramatically enriched, such as cellular respiration, single-organism carbohydrate catabolic, aerobic respiration, oxoacid metabolic, carboxylic acid metabolic, ribose phosphate metabolic and hydrogen transport, which indicated the potential regulation role of Khib in carbohydrate metabolism energy production.

**Figure 4:**
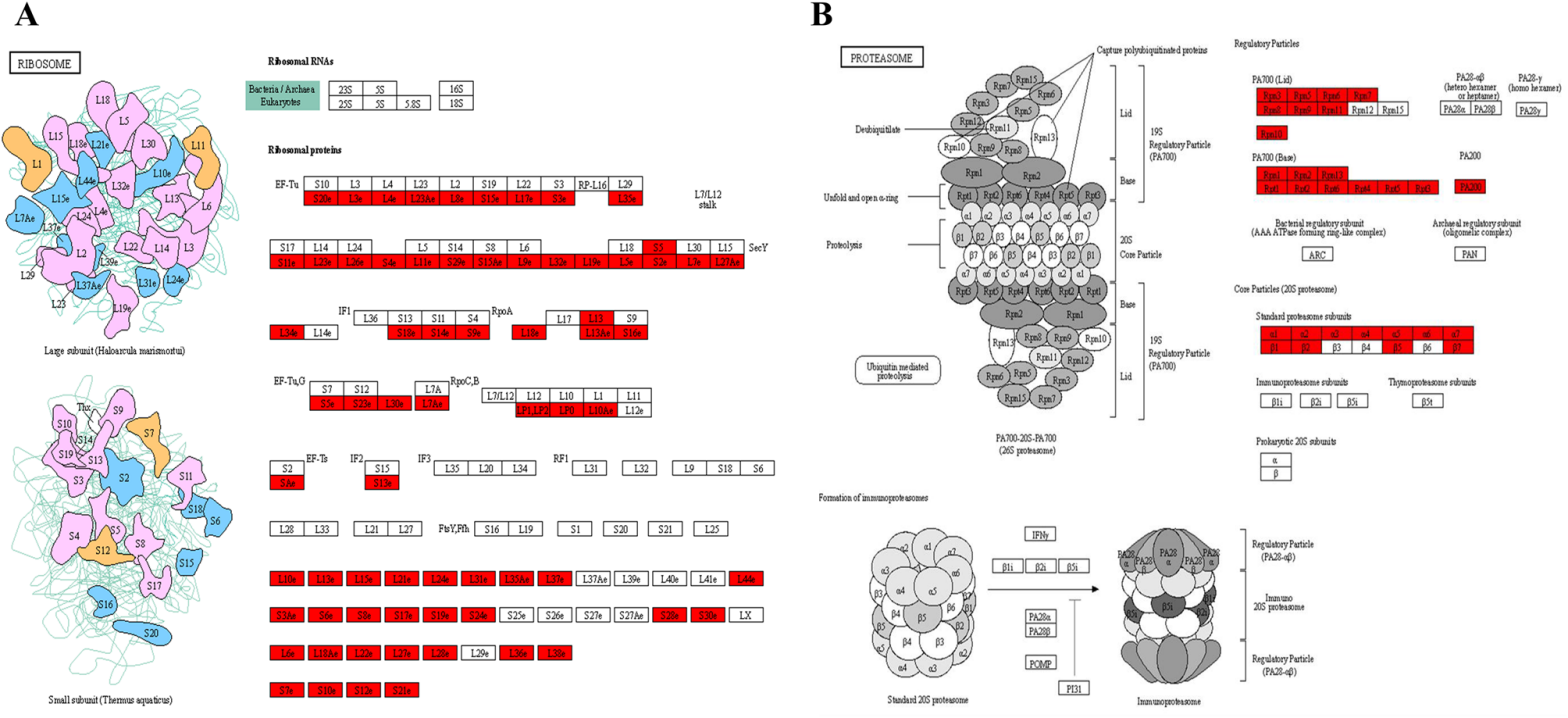
Representative significantly enriched protein metabolism related KEGG pathways. (**A**) Ribosome. (**B**) Proteasome. The Khib proteins are labeled in red.

In the KEGG pathway enrichment analysis (Fig. 3B and Table S2), consistent with the dramatically enriched ribosome and protein metabolism related terms in the category of molecular function and biological process (Fig. 3A), we observed the most significantly enriched pathway was ribosome and the third dramatically enriched pathway was proteasome. As shown in Fig. 4A, both the large subunit and small unit of ribosomal protein were Khib modified with multiple sites. All the three major domains of proteasome complex also exhibited many Khib sites (Fig. 4B). Protein Khib modification may exert a critical role in both protein biosynthesis and protein degradation. In addition, the enriched amino acid metabolism related pathways including alanine, aspartate, glutamate, cysteine, methionine, glycine, serine, threonine, arginine, valine, leucine and isoleucine metabolism provided supplementary evidence to this viewpoint.

**Figure 5:**
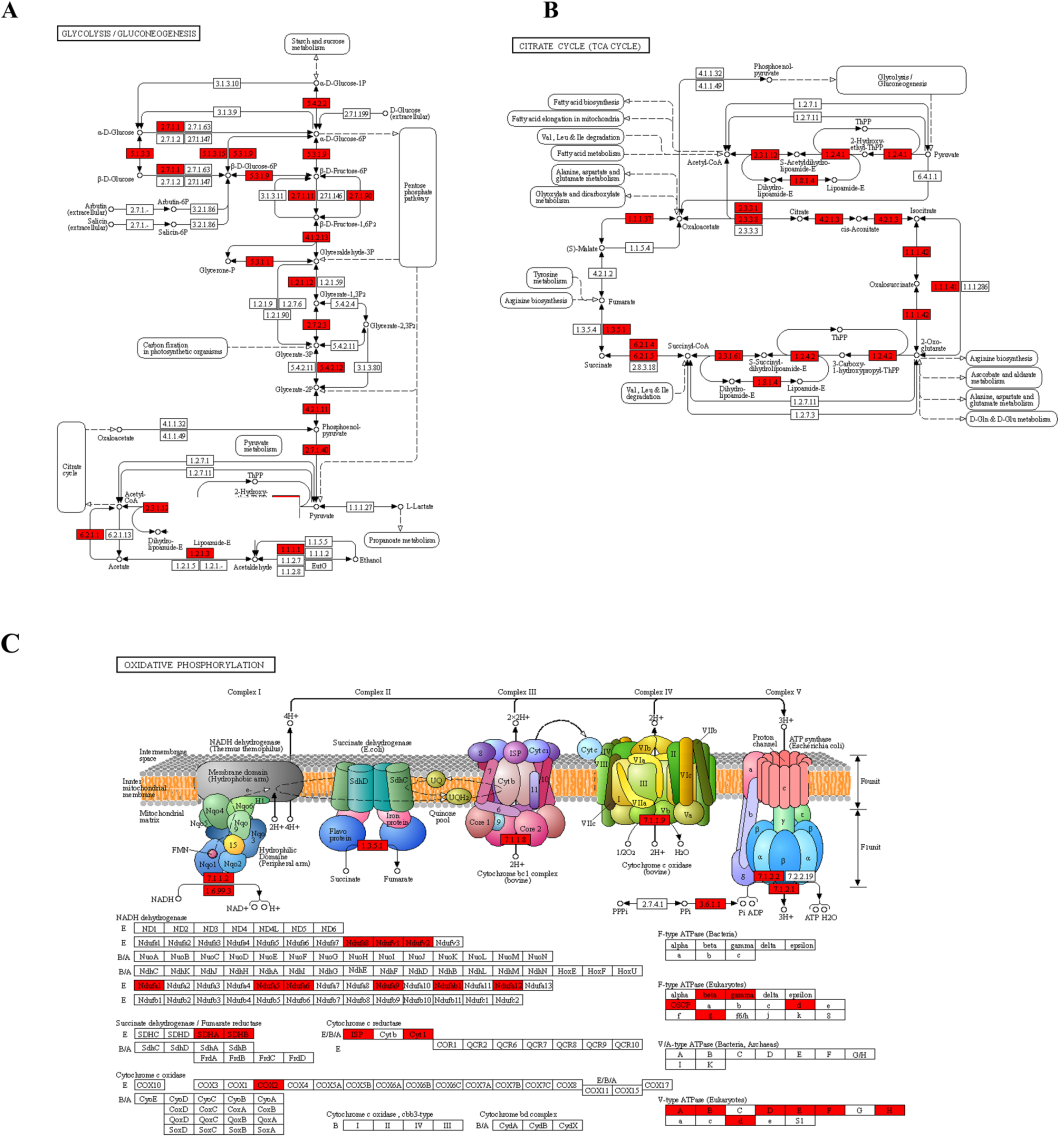
Representative significantly enriched carbohydrate metabolism and energy production related KEGG pathways. (**A**) Glycolysis/gluconeogenesis. (**B**) TCA cycle. (**C**) Oxidative phosphorylation. The Khib proteins are labeled in red.

Many carbon metabolism and energy production related pathways were also markedly enriched in the pathway analysis (Fig. 3B) including TCA cycle, glycolysis/gluconeogenesis, 2-Oxocarboxylic acid metabolism, pyruvate metabolism, pentose phosphate pathway, fatty acid degradation and oxidative phosphorylation. The representative pathways most directly to carbon metabolism and energy production; glycolysis/gluconeogenesis, TCA cycle and oxidative phosphorylation were shown in Fig. 5. Obviously, almost all the major members of the enzymes participating glycolysis/gluconeogenesis and TCA cycle were Khib modified (Fig. 5A,B), such as aconitate hydratase, malate dehydrogenase, isocitrate dehydrogenase, transketolase, succinate–CoA ligase, succinate dehydrogenase, pyruvate dehydrogenase complex, citrate synthase, glyceraldehyde-3-phosphate dehydrogenase, phosphoglycerate kinase, pyruvate kinase, fructose-bisphosphate aldolase, 6-phosphofructokinase, aldehyde dehydrogenase, glucose-6-phosphate isomerase and 2,3-bisphosphoglycerate-independent phosphoglycerate mutase. In addition, all the five protein complexes consisting electron transfer chain of oxidative phosphorylation exhibited various Khib sites on some subunits and protein components (Fig. 5C), implying Khib on the complexes probably influenced the electron and/or hydrogen transfer in the chain and final ATP synthesis process. Combing the markedly enriched carbon metabolism cell respiration and energy production related biological processes in biological process analysis (Fig. 3A); we infer protein Khib may play an important role in carbon metabolism and energy production activity adjust and control.

**Figure 6:**
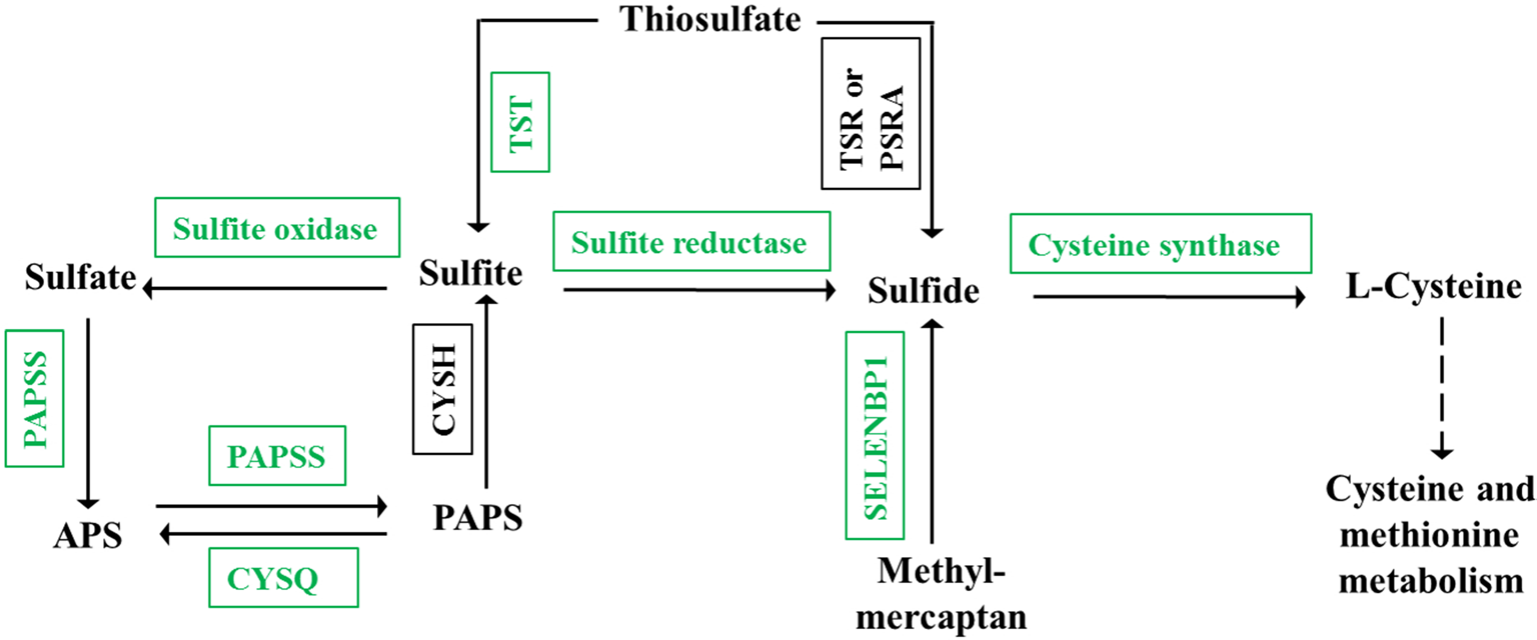
Khib regulated sulfur assimilation and metabolism. The Khib modified enzymes are indicated in green. APS: adenosine 5′-phosphosulfate; PAPS: adenosine 3′-phosphate 5′-phosphosulfate; PAPSS: Sulfate adenylyltransferase; CYSH: phosphoadenosine phosphosulfate reductase; CYSQ: 3′(2′),5′-bisphosphate nucleotidase, SELENBP1:Selenium-binding protein 1, methanethiol oxidase; TST: thiosulfate/3-mercaptopyruvate sulfurtransferase; TSR: thiosulfate-dithioerythritol sulfurtransferase; PSRA: thiosulfate reductase/polysulfide reductase chain A.

It’s noticeable that sulfur metabolism was significantly enriched (Fig. 3B) in the KEGG analysis, implying Khib modification occurred in some sulfur assimilation and transformation related proteins. The involved proteins were shown Fig. 6 and Table S2.

### Comparison of Khib profile among various plant species

Previous studies have reported the Khib profile analysis in rice and *Physcomitrella patens*^13,14^. We compared our data with the Khib profile data in rice and Physcomitrella patens through BLAST analysis, with the purpose of revealing the specificity of Khib in wheat root tissues. As shown in Fig. S3A, only 60 shared Khib sites were found among these three species while as many as 5039 Khib sites (Table S3) were specifically detected in wheat root. KEGG enrichment analysis (Fig. S3B) of the corresponding wheat root specific distributed Khib proteins indicated these Khib modified sites and proteins were dramatically enriched into ribosome pathway with the largest enriched protein number, implying Khib possibly affected ribosome activity and protein synthesis related processes in growing wheat root. Other significant enriched pathway included proteasome, RNA transport, TCA and oxidative phosphorylation.

### Comparison of Khib, Kac and Ksucc in wheat

The systematic Kac and Ksucc screening at proteomics level have been conducted in wheat^17,19^. We compared our lysine 2-hydroxyisobutyrylome data with the acetylome and succinylome data, to find the common or special modification sites for each other. As the result indicated (Fig. S4A), only three sites (Q332R4, K486; Q8LRM8, K84; W5EAP3, K151) could be modified by these three modifications. In addition, Khib shared 20 substrate sites with Ksucc and 81 substrate sites with Kac. As to the wheat root Khib specifically modification sites, 5866 potential substrate sites were observed (Table S4). KEGG pathway result (Supplementary Fig. S4B) showed the substrates of wheat specific Khib modified sites and proteins were mainly involved in protein metabolism related pathways including ribosome, protein processing in endoplasmic reticulu, RNA transport and proteasome. Besides, two energy production related pathways, TCA and oxidative phosphorylation, were also significantly enriched.

## Discussion

### Khib is a widespread modification in wheat root

Khib is a novel discovered PTM in living organisms^2^. Previous studies have reported several system Khib analyses at proteomics level in some species and tissues with the purpose of decipher the potential role of Khib in diverse cellular events and biological processes^9,12,20^. In the field of plant biology, system Khib identification at proteomics level has only been performed in rice seed tissues and *Physcomitrella* patens haploid gametophyte, which detected 9916 Khib sites in 2512 proteins and 11,976 Khib sites in 3001 proteins, respectively^13,14^. In this study, using wheat root as material, we firstly acquired the lysine 2-hydroxyisobutyrylome data in plant root tissues and detected 6328 Khib sites on 2186 proteins, which has enriched the species and scope of Khib studies in plant kingdom as well as in biology. The variance of the identified Khib sites and proteins may be related to several factors, such as plant species, tissues, life stages and plant treatment conditions.

Previous studies in *Physcomitrella patens* and developing rice seed have shown diverse biological process related proteins were Khib modified, and catalyzing and binding related proteins were the primary targets of Khib modification^13,14^, which was consistent with our study. Besides, both studies have shown that cytoplasm and chloroplast were the main subcellular locations where Khib proteins distributed^13,14^. Our study also found cytoplasm was the major place distributed with Khib proteins while chloroplast located proteins were rare, which may be related with the plant parts and tissues (root) we used. In summary, Khib is a widespread modification in wheat root.

### Khib influenced proteins and amino acid metabolism in wheat root

In the enrichment analysis of these Khib proteins (Fig. 3), the result showed that protein metabolism may be influenced by Khib, especially the protein biosynthesis process and ribosome activity. Previous studies in various species including rice, *Physcomitrella patens*, *Proteus mirabili*s, yeast and A549 cells also obtained some markedly enriched protein biosynthesis and/or ribosome related GO terms and/or KEGG pathways; mover, ribosome was even the top significantly enriched GO terms or pathways in some species^2,10,13,14,20^. Protein Khib may be a conservative regulation pattern of protein biosynthesis and ribosome activity. Our study is another evidence of this concept. What’s noticeable was that proteasome, the well-known cellular structure which responses for protein degradation, was the third significantly pathway. It seemed conflict to the most significantly ribosome. The Khib influenced proteasome pathway in previous studies is relatively less, and it has been reported in rice seed and *Toxoplasma gondii* Parasites^12,13^. Protein Khib perhaps participated in the degradation of damaged or protein. Our findings expanded the recognition of Khib influenced protein degradation. We infer Khib probably exerts a coordinating and equilibrating role in proteins synthesis and protein degradation and keep the protein metabolism in a dynamic balance state in growing wheat root. Previous studies have demonstrated that Khib could induce steric bulk, protein charge states change and hydrogen bond formation effects which influence the interaction between bio-macromolecules, and plays a protein function regulation role^2,21^. The diverse Khib sites on the subunits and domains of the two functional complexes (Fig. 4A,B) perhaps affected the interactions among these submits and domains, and performed a role of complex assembling and disassembling adjust and control. Consequently, the biological activity of these complexes was influenced. However, deeper structure and activity experiments are needed to convince this assumption.

Apart from the dramatically enriched protein metabolism related GO terms and pathways, some pathways concerned the metabolism of diverse amino acids were also significantly enriched (Fig. 3B) and as many as 12 different types of amino acids were involved, implying the potential amino acid regulation role of Khib. Previous Khib studies in *Physcomitrella patens*, rice and yeast also observed some amino acid related pathways while the involved amino acid types was relative less^2,13,14^. It well known that amino acids were the structure unit of protein and the substrates of protein biosynthesis. Khib may be actively participated in amino acid metabolism regulation in wheat root development, and further influenced the protein biosynthesis.

### Khib regulated carbohydrate metabolism and energy production in wheat root

Glycometabolism and TCA cycle based Oxidative phosphorylation are of great importance in various wheat life activities including root growth and development as they serve as the dominating material and energy supplier^22,23^. The dramatically enriched carbohydrate metabolism and energy production related GO terms and pathways, especially glycolysis/gluconeogenesis, TCA cycle and oxidative phosphorylation pathway (Figs. 3 and 5), suggested Khib possibly participated in these processes and the role of Khib in carbohydrate metabolism and energy production should be deeper illustrated. Previous study in HCT116 cell lines identified 5 Khib modified glycolytic enzymes^24^. In addition, in yeast, it has been reported a large number of glycolysis/gluconeogenesis related proteins are Khib modified^9^. Our study observed the similar phenomenon in wheat root (Fig. 5A), implying Khib may also play a role in glycolysis/gluconeogenesis regulation in growing root tissues in wheat. In plant species, the study in developing rice seed have reported that the majority enzymes participated in glycolysis/gluconeogenesis, pyruvate metabolism, pentose phosphate pathway, TCA cycle and Oxidative phosphorylation were Khib modified^13^. Consistent with the study in rice, we found the pathways glycolysis/gluconeogenesis, TCA cycle and oxidative phosphorylation were dramatically enriched in the enrichment analysis of the identified Khib proteins in wheat root (Fig. 5). We infer species with relatively close genetic relationship may share similar regulation patterns in Khib mediated glycometabolism metabolism and energy production as the genetic relationship between rice and wheat is relatively close. In addition, some novel enzymes and novel Khib sites were identified in our study, which may facilitate the further illustration of the patterns and mechanisms of Khib influenced carbohydrate metabolism and energy production in wheat root.

### Khib mediated sulfite assimilation and transformation in wheat root

Sulfur is the fourth major essential macronutrient of plant, which is of vital importance for plant growth and diverse physiological functions^25^. Root depended sulfate absorption is the major approach of plant obtaining sulfur; following sulfate absorption, successive reduction and assimilation reaction occurs and the sulfur in sulfate state is finally transformed into cysteine state, upon the catalyzing of diverse sulfur metabolism related enzymes^26,27^.

As shown in Fig. 6, a lot of sulfur assimilation and transformation related enzymes were Khib modified on various sites, such as sulfate adenylyltransferase, sulfite reductase and sulfite oxidase, thiosulfate/3-mercaptopyruvate sulfurtransferase and cysteine synthase. Previous studies in rice and *Physcomitrella patens* have found the Khib modification in cysteine synthase^13,14^. Our finding of these novel sulfur metabolism related enzymes facilitated our understanding of the potential regulation role of Khib modification in sulfur assimilation and transformation in wheat root tissues.

## Conclusion

In the present study, the global Khib analysis in wheat root tissues was firstly studied, which has expanded both the specie and scope of Khib in plant. In sum, 6328 Khib sites corresponding to 2186 proteins were repeatedly identified in three replicates which showed diverse cellular components and subcellular compartments distribution. Function and pathways analysis indicated multiple cellular functions and metabolism pathways were potentially influenced by Khib modification. Protein and amino acid metabolism related process may be regulated by Khib, especially ribosome activities and proteins biosynthesis processes. In addition, carbohydrate metabolism and energy production related pathways were also the preferred metabolism pathways where protein Khib occurred. The activities and stabilities of the enzymes in carbon and energy metabolism may be regulated by Khib modification. Moreover, sulfur metabolism is another important metabolism pathway which could be influenced by Khib in wheat root tissues. Our work could serve as a useful resource for the function and pathway illustration of Khib in wheat root growth, development and root biological, as well as in other species plant root.

## Materials and methods

### Wheat cultivation

The selected wheat (*Triticum aestivum* L.) cultivar was Jimai 44. The geographical location of the wheat cultivation and collection procedure was Shandong Academy of Agricultural Sciences (SAAS) in Jinan (36°42′ N, 117°4′ E; altitude 48 m), Shandong Province, China. The general climate indicators were listed as follows: the average annual temperature was 14.5 °C; the average annual amount of sunshine was 2600 h; the mean rainfall was around 700 mm. In October, 2019 (wheat sowing season in North China), the wheat seed were grown in the pots (diameter 30 cm, height 35 cm) filling with 20 kg soil. The type of soil was Typic-Hapli-Udic Argosols based on Chinese Soil Taxonomy^28^. The soil properties include organic matter content, 12.01 g/kg; total nitrogen, 0.65 g/kg; available phosphorus, 13.3 mg/kg; available potassium, 92.22 mg/kg; and PH, 7.85. The normal fertilizers were applied once accompany sowing. The wheat seedlings were treated with normal water condition in the following 40 days cultivation stage.

### Collection of wheat roots

After 40 days cultivation, the roots from 20 plants in a pot were harvested and washed for three times. Then the clean roots were mixed as one independent biological replicate. After liquid nitrogen frozen, the root tissues were stored at − 80 °C. Three biological replicates were collected^29^.

### Permission statement

The experimental design of the whole study including collection of plant specimens has been approved by Crop Research Institute, Shandong Academy of Agricultural Sciences, Ji’nan, P. R. China.

### Protein extraction

The protein extraction was conducted according to previous report with some modifications^30^. Briefly, clean wheat root tissues were grinded into powder in a mortar with liquid nitrogen. Then the powder were transfer to a tube and re-suspended in ice cold extraction buffer (250 mM sucrose, 50 mM Tris–HCl, pH 7.5, 1% Triton X-100, 2 mM EDTA, 3 μM TSA, 50 mM NAM, 10 mM DTT and 1% protease Inhibitor Cocktail). After 5 min sonication on ice, the extraction buffer was mixed with same volume ice-cold Tris buffer phenol (pH 8.0) and agitated for 15 min. After centrifugation at 15,000×*g* for 20 min, transfer the phenolic phase to a new tube and add four volumes of 100 mM ammonium acetate in methanol to the tube and keep the tube at − 20 °C for 12 h. Following centrifugation (15 min, 15,000×*g*), the precipitated protein pellet was rinsed three times with ice-cold acetone, vacuum-dried and stored at − 80 °C.

### Trypsin digestion

The protein pellets were dissolved in 8 M urea through 5 min sonication on ice and then quantified with a 2-D Quant kit (GE Healthcare, America) referring the manufacturer’s instruction. The sampled was firstly incubated with 10 mM DTT at 37 °C for 1 h and then incubated with 25 mM iodoacetamide (IAM) for 0.5 h at room temperature in dark. Four volume TEAB buffer (100 mM) was added to the protein solution to make sure urea concentration less than 2 M. Trypsin was added into the sample at 1: 50 trypsin-to-protein mass ratio for 12 h digestion. Then the second time digestion procedure was performed (1:100 trypsin-to-protein mass ratio, 4 h). Finally, peptide was desalted by Strata X C18 SPE column (Phenomenex) and vacuum-dried.

### HPLC fractionation and affinity enrichment

The dried peptides were re-suspended in buffer A (98% H_2_O, 5 mM NH_4_OH) and then loaded into the column (C18, 5 μm particles, 4.6 mm ID, 250 mm length) in Agilent 1260 HPLC instrument. The LC gradient was initiated at 5% buffer B (80% ACN, 5 mM NH_4_OH) and increased to 30% in 14 min. Then it climbed to 80% in the following 6 min. The resulted 96 fractions were combined into 4 fractions for the system Khib identification.

To separate the modified peptides, an immunoprecipitation affinity enrichment process was carried out with agarose-conjugated anti Khib antibody (WM502, Micrometer Biotech, China)^31^. Peptides were dissolved in NETN buffer (100 mM NaCl, 1 mM EDTA, 50 mM Tris–HCl, 0.5% NP-40, pH 8.0). The beads were pre-washed with NETN buffer for two times. Then incubate the peptides solution and beads for at 4 °C for 16 h in gentle shaken. Rinse the beads for 4 times with NETN buffer and 2 times with purified water after incubation. Finally, elute the Khib modified peptides with 0.1% Trifluoroacetic acid (TFA) and vacuum dry them.

### LC–MS/MS analysis

Data acquisition was carried out referring previous report with a Q Exactive (Thermo Scientific) mass spectrometer combineing EASY-nLC 1000 UPLC system (Thermo Fisher Scientific)^32^. The dried peptides were re-suspended in sovent A (0.1% FA, 100% H_2_O) and centrifuged at 15,000×*g* for 10 min. Transfer the supernatant to a RP analytical column (Thermo Acclaim PepMap RSLC C18 column, 2 μm, 75 μm × 50 mm) in an EASY-nLC UPLC instrument (Ultimate RSLCnano 3000). The LC flow rate was set at 250 nl/min and the gradient was 2% to 10% solvent B (0.1% FA in 80% ACN) for 6 min, 10% to 20% for 45 min, and to 80% within 4 min then holding at 80% for 1 min.

The detailed data acquisition parameters were included as follows. The acquisition mode was set as Data-dependent acquisition (DDA). Intact peptides were detected at a resolution of 60,000 and MS scan range was 350–1800 Da. Peptides selected for MS/MS analysis using 25% normalized collisional energy (NCE) and the Ion fragments were detected at a resolution of 30,000. The electrospray voltage was set to 2.0 kV, automatic gain control (AGC) was used to prevent overfilling of the Orbitrap and 5E4 ions were accumulated for the generation of MS/MS. The maximum injection time (MIT) was 200 ms. In a scan cycle; one MS scan following 15 MS/MS scans was applied for the top 15 precursor ions collection with 15.0 s dynamic exclusion and ion count of 1E5. LC–MS/MS analysis was performed blindly by Micrometer Biotech Company (Hangzhou, China). The raw data were deposited to ProteomeXchange Consortium^33^ with the accession number PXD020819.

### Database searching

The MaxQuant software with integrated Andromeda search engine (v.1.4.1.2) was used for database searching. Tandem mass spectra were searched against the database of wheat (*Triticum aestivum* L.) in Uniprot (130,673 sequence, October, 2019) concatenated with reverse decoy database. Trypsin/P was specified as the cleavage enzyme and a maximum of 4 missing cleavages was allowed. The mass error was set to 10 ppm for precursor ions and 0.02 Da for fragment ions. Carbamidomethylation on cysteine was specified as fixed modification. Variable modification was defined as oxidation on methionine and 2-hydroxyisobutyrylation on both lysine and N-terminal of protein. False discovery rate (FDR) thresholds were specified at 1% and the minimum peptide length was set to 7. All of the other parameters were set to default.

### Bioinformatics analysis

The function classification and enrichment analysis was performed based on gene ontology (GO) annotation and Kyoto Encyclopedia of Genes and Genomes (KEGG) database^34,35^. The protein ID was converted to UniProt ID and then mapped to GO IDs by protein ID. The proteins were classified by GO annotation on the category of biological process, cellular compartment, and molecular function. Subcellular localization analysis was performed with Wolfpsort subcellular localization predication software^36^. GO, KEGG pathway and domain enrichment analyses were carried out by DAVID tool with corrected p-value below 0.05^37^. The two-tailed Fisher’s exact test was applied to check the Khib proteins. Multiple testing correction was performed using the Benjamini–Hochberg false discovery rate (FDR) control method^37^. The images of the significantly pathways were obtained from KEGG.

### Western blotting

Proteins were separated using 12% SDS-PAGE gel, and then transferred into a PVDF membrane. After blocking for 1 h with 5% skim milk, the membrane was incubated with pan anti-2-hydroxyisobutyryllysine antibody (WM501, rabbit polyclonal antibody, Micrometer Biotech, China) overnight in dilution 1:1500. The membrane was washed three times to remove the unbinding primary antibody and then incubated with a secondary horseradish peroxidase-conjugated goat-anti-rabbit antibody at for 2 h (1:20,000 dilution)^13^.

### In vitro 3-hydroxyisobutyryl-CoA treatment and assay

In vitro 3-Hydroxyisobutyryl-CoA treatment for the extracted proteins was performed referring previous report^8^. Briefly, 20 μg proteins and 10 μM 3-Hydroxyisobutyryl-CoA were added into the reaction buffer containing 50 mM Tris-CI, pH 8.0, 100 nM TSA, 10% glycerol, 5 mM Nicotinamide, 1 mM DTT, 0.1 mM EDTA and 1 × proteinase inhibitor mixture. The reaction mixtures were incubated at 30 °C for 1 h. After treatment, the mixture was fetched to perform the western blotting assay with pan anti-2-hydroxyisobutyryllysine antibody (WM503, mouse monoclonal antibody, Micrometer Biotech, China). The 3-Hydroxyisobutyryl-CoA was synthesized with previous report^38^.

## Supplementary information

Supplementary information.
